# Rethinking the Origin of Auxin Biosynthesis in Plants

**DOI:** 10.3389/fpls.2015.01093

**Published:** 2015-12-02

**Authors:** Meng Ke, Yuyu Zheng, Ziqiang Zhu

**Affiliations:** ^1^School of Life Sciences, Tsinghua UniversityBeijing, China; ^2^College of Life Sciences, Nanjing Normal UniversityNanjing, China

**Keywords:** auxin, TAA1, charophytes, ethylene, *Klebsormidium flaccidum*

Auxin is the first identified and essential phytohormone for plant development and survival (Lee and Cho, 2013; Locascio et al., 2014). The synthesis of naturally occurring auxin indole-3-acetic acid (IAA) in plants can be divided into two categories: tryptophan (Trp)-dependent and Trp-independent pathways (Tivendale et al., 2014). Genetic studies have demonstrated the Trp-dependent pathway as the main route for auxin biosynthesis in plants. Among the four distinctive biosynthesis routes downstream of Trp, the indole-3-pyruvic acid (IPA) pathway is the most crucial one. TRYPTOPHAN AMINOTRANSFERASE OF ARABIDOPSIS 1 (TAA1) catalyzes the formation of IPA from L-Trp and flavin-containing monooxygenase enzymes (YUCCA) further transform IPA into IAA. Although, these auxin biosynthesis routes are well illustrated in higher plants, the origin of auxin biosynthesis in plants is still unclear.

Within the last 1 year, four different Opinions or Letters published in the journal Trends in Plant Science argue whether or not Trp-dependent auxin biosynthesis in plants originates from the charophyte species (Huang et al., 2014; Wang et al., 2014; Yue et al., 2014; Turnaev et al., 2015), which are freshwater plants closely related to land plants during evolution. The major discrepancy among these arguments lie in whether or not charophytes have functional TAA1 (Stepanova et al., 2008; Tao et al., 2008).

Being key players in auxin biosynthesis, TAA1 and YUCCA have been adopted as queries for searching the origin of auxin biosynthesis. In the search, Wang et al. identified one TAA1 homolog (kfl00051_0080) and one YUCCA homolog (kfl00109_0340) in *Klebsormidium flaccidum* (Klebsormidiales, Charophyta) with the help of the recently released *K. flaccidum* draft genome sequence (Hori et al., 2014). They performed phylogenetic analysis and stated that auxin biosynthesis originates from charophytes. However, their conclusion quickly came under attack due to two major issues. Firstly, TAA1 can only be found in *K. flaccidum*, but not in the other four charophyte species (*Mesostigma viride, Coleochaete orbicularis, Spirogyra pratensis*, and *Nitella mirabilis*), according to a recent report on charophyte transcriptome data (Ju et al., 2015). Secondly, phylogenetic analysis and protein structure prediction results show that *K. flaccidum* TAA1 (KfTAA1) is more similar to alliinase but not Arabidopsis TAA1 (AtTAA1) (Huang et al., 2014; Turnaev et al., 2015).

Nevertheless, we think it is still too early to conclude that auxin biosynthesis does not originate from charophytes. Firstly, although *TAA1* homologs have not been identified in other charophyte species, it does not necessarily mean that they do not contain *TAA1*. Since interpretation of transcriptomic data highly depends on the sample collection stage, the absence of *TAA1* in other species can very likely be attributed to incorrect developmental or treatment stage.

Secondly, although Turnaev et al. predicted the KfTAA1 three dimensional structure with I-TASSER software (Turnaev et al., 2015), we performed a homology modeling for structure comparison, using AtTAA1 [Protein Data Bank (PDB, http://www.rcsb.org) ID 3BWO: A] as a template. We input the AtTAA1 template and the C-terminus of KfTAA1 sequence in the automated protein structure homology-modeling server SWISS-MODEL (http://www.swissmodel.expasy.org) for calculation. The active site of the C-terminus of KfTAA1 highly resembles that of AtTAA1 (Figure 1), which suggests that Trp could be a potential substrate for KfTAA1 in *K.flaccidum*. Of course, without solid biochemical data, nobody can conclude that KfTAA1 is the *bona fide* Trp aminotransferase for IPA synthesis.

**Figure 1:**
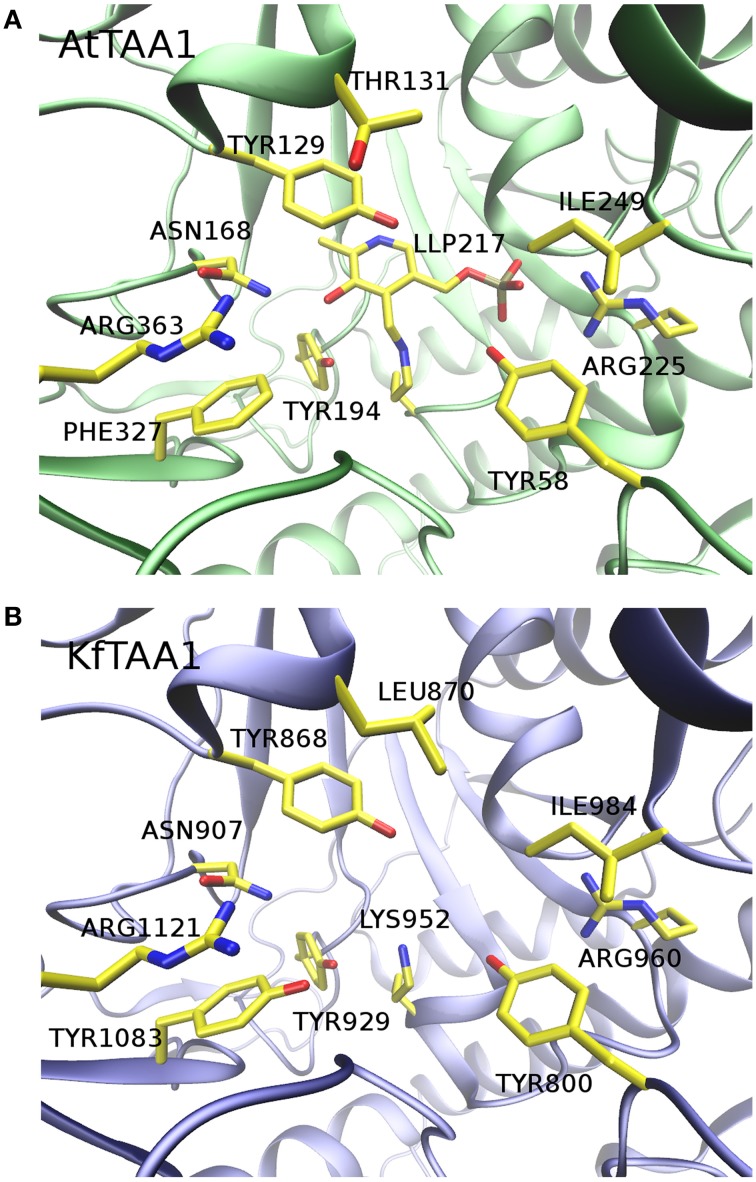
**Comparison of AtTAA1 crystal structure to KfTAA1 homology model**. **(A)** Active site of AtTAA1 (PDB ID 3BWO: A). The cofactor N'-pyridoxyl-lysine-5′-monophosphate (LLP) and crucial residues surrounding the active site are indicated. **(B)** Active site of KfTAA1 model. Without modeling LLP, residues in equivalent positions as showed in **(A)** are highlighted.

Last but not least, we have to rethink the origin of auxin biosynthesis not only based on sequence analysis, but also take its biological functions into account, especially in the current stages with limited experimental evidence. Recent phylogenetic, genetic, and biochemical studies on the origin of ethylene in plants demonstrate that ethylene originates from charophytes (Ju et al., 2015). However, it is widely accepted that ethylene and auxin actions are intertwined, from biosynthesis to signal transduction. For example, ethylene inhibits root growth elongation, which is caused by the induction of auxin biosynthesis (Stepanova et al., 2007, 2008). Plants impaired in *TAA1* in Arabidopsis are largely insensitive to ethylene in root growth inhibition (Stepanova et al., 2008). Another, hallmark phenotype of ethylene is the induction of exaggerate apical hook formation in etiolated seedlings, which also requires auxin transport and signaling to cause the unequal cell elongation rates in the two sides of hypocotyl (Abbas et al., 2013; Mazzella et al., 2014). Since it has been proven that ethylene regulates cell elongation in charophytes, it would be very interesting to further dissect whether or not auxin participates in ethylene actions in charophytes. There will be two possibilities: one is that auxin exists in charophytes but is not involved in ethylene functions, which implies that auxin is adopted in ethylene function after the colonization of land; the other possibility is that auxin do participate in ethylene signaling. However, recent multispecies genome-wide analysis results show that nearly all the orthologs of auxin biosynthesis, transport, and signaling components are already exist in *K. flaccidum* as in higher plants (Wang et al., 2015). Thus, it is not likely that auxin does not act with ethylene in charophytes as we proposed in the first situation. Based on these uncertain scenarios, further investigations on auxin signaling origin and its interaction with ethylene signaling (and/or other phytohormones) will be promising scientific questions for understanding how and when hormone signal transductions are evolved and diverged.

Collectively, we do not think it is proper to conclude that auxin biosynthesis in plants does not originate from charophytes. The bioactivities of KfTAA1 and functions of auxin must also be considered together with traditional phylogenetic analysis. The hormone research community should switch their emphasis on sequence similarities to wet experiments.

1. One possible reason for the lacking of *TAA1* in charophyte transcriptomic data is that the expression of *TAA1* is under environmental stimuli control. According to the knowledge from Arabidopsis, it is urgent to re-examine *TAA1* expression under ethylene treatment in charophyte species.
2. As *de novo* gene synthesis becomes both simpler and cheaper, it will become feasible to directly clone charophyte *TAA1* and test its enzymatic activities *in vitro* and perform genetic complementation analysis to see if charophyte *TAA1* can somewhat complement Arabidopsis *taa1* mutant phenotypes.
3. Given that the technology for agrobacterium based stable transformation in charophytes is established very recently (Sørensen et al., 2014), it will be practicable to obtain *KfTAA1* mutants via RNA interference (RNAi) or CRISPR/Cas9 approaches for determining KfTAA1 functions in charophytes.
4. Chemical inhibitors are useful tools for studying auxin origins and auxin-ethylene interactions. It has been reported that L-kynurenine (Kyn) is an Arabidopsis TAA1 inhibitor (He et al., 2011), while 4-biphenylboronic acid (BBo) and 4-phenoxyphenylboronic acid (PPBo) are YUCCA inhibitors (Kakei et al., 2015). As we know that ethylene promotes cell elongation in charophytes, how about its phenotype in the presence of these inhibitors? If KfTAA1 has enzymatic activity, can Kyn inhibit its activity as it did in Arabidopsis?

After systematic investigations, we believe that the plant hormone community will ultimately elucidate the truth in the near future.

## Author contributions

MK modeled protein structure; YZ analyzed data; ZZ designed research and wrote the paper.

## Funding

Researches in the Zhu laboratory are supported by the National Natural Science Foundation of China (31470375), the Natural Science Foundation of Jiangsu Province (BK20140919), the Natural Science Foundation of the Jiangsu Higher Education Institutions of China (14KJB180014) and the Priority Academic Program Development of Jiangsu Higher Education Institutions.

### Conflict of interest statement

The authors declare that the research was conducted in the absence of any commercial or financial relationships that could be construed as a potential conflict of interest.
